# Genome-wide analyses identify *NEAT1* as genetic modifier of age at onset of amyotrophic lateral sclerosis

**DOI:** 10.1186/s13024-023-00669-6

**Published:** 2023-10-23

**Authors:** Chunyu Li, Qianqian Wei, Yanbing Hou, Junyu Lin, Ruwei Ou, Lingyu Zhang, Qirui Jiang, Yi Xiao, Kuncheng Liu, Xueping Chen, TianMi Yang, Wei Song, Bi Zhao, Ying Wu, Huifang Shang

**Affiliations:** Department of Neurology, Laboratory of Neurodegenerative Disorders, West China Hospital, National Clinical Research Center for Geriatric, Sichuan University, Chengdu, China

**Keywords:** Amyotrophic Lateral Sclerosis, Age at onset, GWAS, Genetic modifiers, *NEAT1*

## Abstract

**Background:**

Patients with amyotrophic lateral sclerosis (ALS) demonstrate great heterogeneity in the age at onset (AAO), which is closely related to the course of disease. However, most genetic studies focused on the risk of ALS, while the genetic background underlying AAO of ALS is still unknown.

**Methods:**

To identify genetic determinants influencing AAO of ALS, we performed genome-wide association analysis using a Cox proportional hazards model in 2,841 patients with ALS (N_discovery_ = 2,272, N_replication_ = 569) in the Chinese population. We further conducted colocalization analysis using public cis-eQTL dataset, and Mendelian randomization analysis to identify risk factors for AAO of ALS. Finally, functional experiments including dual-luciferase reporter assay and RT-qPCR were performed to explore the regulatory effect of the target variant.

**Results:**

The total heritability of AAO of ALS was ~ 0.24. One novel locus rs10128627 (*FRMD8*) was significantly associated with earlier AAO by ~ 3.15 years (P = 1.54E-08, beta = 0.31, SE = 0.05). This locus was cis-eQTL of *NEAT1* in multiple brain tissues and blood. Colocalization analysis detected association signals at this locus between AAO of ALS and expression of *NEAT1*. Furthermore, functional exploration supported the variant rs10128627 was associated with upregulated expression of *NEAT1* in cell models and patients with ALS. Causal inference suggested higher total cholesterol, low-density lipoprotein, and eosinophil were nominally associated with earlier AAO of ALS, while monocyte might delay the AAO.

**Conclusions:**

Collective evidence from genetic, bioinformatic, and functional results suggested *NEAT1* as a key player in the disease progression of ALS. These findings improve the current understanding of the genetic role in AAO of ALS, and provide a novel target for further research on the pathogenesis and therapeutic options to delay the disease onset.

**Supplementary Information:**

The online version contains supplementary material available at 10.1186/s13024-023-00669-6.

## Background

Amyotrophic lateral sclerosis (ALS) is a progressive neurodegenerative disorder characterized by upper and lower motor neuron loss which ultimately leads to respiratory failure [1]. Though the peak age at onset (AAO) of ALS is 45–49 years for women and 55–59 years for men [2], individuals of any age could be affected. The AAO of ALS is a key phenotypic feature associated with variation in the motor and cognitive phenotypes [3], disease progression, prognosis, and survival [4, 5]. Identification of risk factors influencing AAO of ALS could help better understand the disease etiology and provide clinical guidance for patients and clinicians. However, the mechanisms underlying the heterogeneity in AAO of ALS are complex and still poorly understood.

Epidemiological studies have suggested several clinical factors like sex, family history, and physical activity might influence the AAO of ALS [3, 6]. However, these factors explain only a modest proportion of the variance in AAO of ALS. In recent years, along with the burgeoning genetic research on ALS, growing evidence demonstrated the essential role of genetic background in the AAO of ALS. For example, whole exome sequencing analysis in 89 families with familial ALS and 410 patients with sporadic ALS suggested that multiple rare variants in ALS causative genes like *SOD1* could lead to earlier AAO [7]. Meanwhile, a previous genome-wide association study (GWAS) in the European population identified suggestive association between several genomic regions and AAO of ALS [8]. Identifying genetic modifiers for AAO of ALS could help provide additional insights into the disease mechanisms and find out therapeutic options that may postpone the disease onset. However, most genetic studies mainly focused on the risk of ALS, while the genetic risk factors affecting AAO of ALS are mostly unknown, especially in the Asian population.

In this context, we carried out a GWAS in a large ALS cohort to explore genetic modifiers for AAO of ALS. We identified a novel locus rs10128627 (*FRMD8*) which was significantly associated with earlier AAO of ALS.

## Methods

### Participants

The participants comprised 2,841 unrelated Chinese patients with ALS recruited from the Department of Neurology of West China Hospital of Sichuan University (N_discovery_ = 2,272, N_replication_ = 569). The patients were diagnosed by neurologists specializing in ALS according to the El Escorial revised criteria [9]. AAO was defined as the age when the patient presented with upper or lower motor neuron signs. West China Hospital approved the study, and all participants have signed informed consent.

### Genotyping

Genomic DNA was extracted from peripheral blood leukocytes and then genotyped on the Illumina Infinium Asian Screening Array-MD v1.0 for a total of ~ 0.66 million single nucleotide polymorphisms (SNP) [10]. Imputation was performed in Michigan Imputation Server with the reference panel Genome Asia Pilot [11]. Imputed SNPs with *r*^*2*^ < 0.8 and genotype calls with genotype quality < 0.8 were removed.

### Quality control

Individuals with mismatched sex or a call rate of less than 95% were removed. Related individuals assessed by IBD analysis using Plink were removed. SNPs with missingness > 0.05 or out of Hardy-Weinberg Equilibrium (P < 1E-06) or with minor allele frequency (MAF) < 0.01 were excluded. To remove potential outliers affecting the regression model, patients with AAO < 20 years or AAO > 89 years (around ± 3 standard deviations from the mean) were excluded. Ancestry outliers identified by the principal component analysis (PCA) based on the 1000 Genomes Project haplotypes were removed.

### Genome-wide survival analysis

We first performed genome-wide survival analysis using a Cox proportional hazards model for AAO of ALS in the discovery cohort, adjusting for sex and the first three principal components. Then we applied the same model in the replication cohort, and combined the results from both stages into a meta-analysis using Plink under a fixed-effect model. Log-rank test was used to assess the difference of AAO between categories. The significant level was set as P = 5E-08 at genome-wide, and P = 1E-06 was set as the suggestive significance level.

We next conducted gene-based association analysis using MAGMA (Multi-marker Analysis of GenoMic Annotation) with default parameters to integrate association signals from the variant level into the gene level [12]. The subjects from the East Asian population in the 1000 Genomes Project (Phase 3) were used for the linkage disequilibrium (LD) reference. P value was adjusted by Bonferroni correction according to the number of tested genes.

Polygenic risk score (PRS) has been utilized in estimating the genetic risk of developing specific diseases. In the current study, we evaluated the correlation between AAO and PRS of ALS risk using a Cox proportional hazards model. When calculating PRS of risk with PRSice [13], risk allele dosages scaled by beta coefficients were summed across genome-wide SNPs from the largest GWAS on ALS risk so far [14]. Considering the genetic heterogeneity across ethnicities, we further calculated PRS using summary statistics from a meta-analysis involving two earlier GWAS on risk of ALS in the Asian population [15, 16]. Meta-analysis was performed using Plink with a random-effect model. PRS was converted to Z scores to make the scale of analyses more easily interpretable. To further test the association between ALS of AAO and each risk variant of ALS, we performed survival analysis using a Cox proportional hazards model and linear regression among the targeted risk SNPs of ALS [14]. Statistical analyses were performed in R v3.5.3.

Furthermore, to explore the potential utility of PRS of AAO in clinical trials, we calculated PRS of AAO based on the results from the current GWAS to predict early-onset ALS (AAO < 45 years). Predictability was estimated with receiver operating characteristic (ROC) curves. Considering that overfitting might happen when testing the predictive performance in the same cohort for which the model was trained and thus might bias the results, we tested the performance of the model in samples from the replication stage using summary statistics of AAO of ALS derived from the discovery stage.

### Functional interpretation of significant variants

Since most loci identified by GWAS are thought to regulate gene expression, we screened the significant SNPs from our GWAS for cis-eQTL signals in GTEx [17] and QTLbase [18]. Then we searched GeneHancer for gene enhancers [19], which includes known enhancers and their potential gene targets assessed by functional studies. Furthermore, we evaluated the probability of colocalization between influence on AAO of ALS and regulation of gene expression with colocalization analysis using the coloc package [20]. Cis-eQTL dataset for brain and whole blood from GTEx was utilized [21]. Loci with a posterior probability of hypothesis 4 (PP4) of 0.75 or more were considered colocalized due to a single shared causal variant.

### Dual-luciferase reporter assay

The pGL3-promoter vector was from Promega, and we modified the vector by replacing the original SV40 promoter in the vector with the promoter of *NEAT1* (Additional file 3). About 1000 bp including the lead SNP with reference allele (wildtype) or risk allele (variant) were cloned into the pGL3-promoter vector, which contained the *NEAT1* promoter upstream of the firefly luciferase reporter gene. Hela cells were cultured as described previously [22], and plated in 24-well plates and cotransfected with 0.5 µg of pGL3-promoter-WT or pGL3-promoter-MT and 0.4 µg of pRL-TK vector (Promega) as an internal control by using jetPRIME® transfection reagent (Polyplus-Transfection, France). Moreover, the pGL3-basic vector and pGL3-*NEAT1-*promoter vector were transferred as a systemic control. Luciferase activity was measured 48 h after transfection and the assay was conducted according to the standard protocol of the Promega Dual-Luciferase Reporter Assay System. A minimum of three independent experiments were performed, each including three technical replicates.

All quantitative results of cell assays were presented as the mean and standard error of the mean (SEM) from at least three independent experiments and analyzed with GraphPad Prism 8.0 software. Significant statistical differences between two groups were determined by the two-tailed unpaired Student’s t test and for three or more groups, one-way ANOVA (followed by a Dunnett post hoc test) was used for the statistical analysis. P-values of 0.05 (∗), 0.01 (∗∗) and 0.001 (∗∗∗) were assumed as the level of significance for the statistical tests carried out.

### RNA extraction and RT-qPCR assay

We analyzed the RNA expression level of the target gene in the patients with reverse transcription-quantitative PCR (RT-qPCR). Total RNAs were extracted from peripheral blood mononuclear cells (PBMCs) using Trizol reagent (Invitrogen, USA). cDNA was synthesized from 2 µg of total RNA according to the manufacturer’s instructions (Takara, Japan). RT-qPCR was performed using the QuantStudio 3 system (Thermo Fisher Scientific, USA), and the relative gene expression was normalized to the internal control *ACTB*. Primer sequences of target genes were as follows, and data analysis was performed using the 2^−ΔΔCt^ method.

**Table:** 

Gene	Forward (5’-3’)	Reverse (5’-3’)
*ACTB*	AGAGCCTCGCCTTTGCC	GGGGTACTTCAGGGTGAGGA
*NEAT1*	GATCTTTTCCACCCCAAGAGTACATAA	CTCACACAAACACAGATTCCACAAC

### Mendelian randomization analysis

To identify risk factors influencing AAO of ALS, we further performed Mendelian randomization (MR) analysis to evaluate causal inference among 21 exposures for which there was prior evidence for an association with ALS. These include seven behavioral-related traits: body mass index, years of schooling (educational attainment), alcoholic drinks per week, age of smoking initiation, cigarettes per day, days per week of moderate physical activity and days per week of vigorous activity; three blood pressure traits (coronary artery disease, diastolic blood pressure, and systolic blood pressure); six immune system traits (basophil, eosinophil, lymphocyte, monocyte, neutrophil, and white blood cell counts) and C-reactive protein; and four lipid traits (HDL cholesterol, LDL cholesterol, total cholesterol (TC) and triglyceride levels) (Supplementary Table 1). SNPs that passed the genome-wide significance threshold (P < 5E-08) were chosen as instrumental variables, which were then clumped based on the 1,000 Genomes Project LD structure [23]. Harmonization was undertaken to rule out strand mismatches and ensure alignment of SNP effect sizes.

We performed a two-sample MR analysis using the random effects inverse variance weighted (IVW) method [24], which was most widely used in MR studies and could provide robust causal estimates under the absence of directional pleiotropy. A P value below 2.38E-03 (0.05/21) was considered statistically significant after the Bonferroni correction. In addition, we conducted comprehensive sensitivity analyses to estimate potential violations of the model assumptions in the MR analysis. We conducted Mendelian randomization pleiotropy residual sum and outlier (MR-PRESSO) analysis and leave-one-out analysis to detect outlier instrumental variables. Outlier instrumental variables identified by the MR-PRESSO analysis were removed step-by-step to reduce the effect of horizontal pleiotropy. Cochran’s Q test was executed to check heterogeneity across the individual causal effects. MR-Egger regression was performed to evaluate the directional pleiotropy of instrumental variables. To evaluate the strength of each instrumental variable, we computed the F-statistic of each SNP. The statistical analyses were conducted using R package TwoSampleMR 0.5.5 [25].

## Results

### Demographics and heritability

The final statistical analysis included a total of 2,788 patients after quality control, with 2,230 in the discovery phase and 558 patients in the replication phase. The average AAO (SD) was 54.56 (11.56) with a sex ratio of 1.40 (male/female: 1301/929) in the discovery phase, and the average AAO (SD) was 54.69 (11.33) with a sex ratio of 1.46 (male/female: 331/227) in the replication phase (Supplementary Table 2). The overall distribution of AAO was shown in Supplementary Fig. 1. Generally, males tend to have later AAO than females (55.00 vs. 54.00), but the difference was not significant (P = 0.2) (Supplementary Fig. 2). The total heritability was 0.24 (SE = 0.09) estimated using GCTA-GREML with default parameters [26], which was less than the heritability of risk for ALS (~ 0.50) [27, 28].

### Genome-wide association results

We performed genome-wide association analysis using a Cox proportional hazards model in the discovery and replication phases, and then combined the results from both stages into a meta-analysis. The principal component analysis revealed no descent outlier (Supplementary Fig. 3). The genomic inflation factor λ was 1.020 and 1.025 in two stages, suggesting minimal bias from population stratification. In the discovery stage, one locus rs6801884 (*SATB1*; *KCNH8*) reached genome-wide significance level (P = 1.81E-11, beta = 0.99, SE = 0.15), and another locus rs10128627 (*FRMD8*) was suggestively significant (P = 3.49E-07, beta = 0.31, SE = 0.06).

**Fig. 1:**
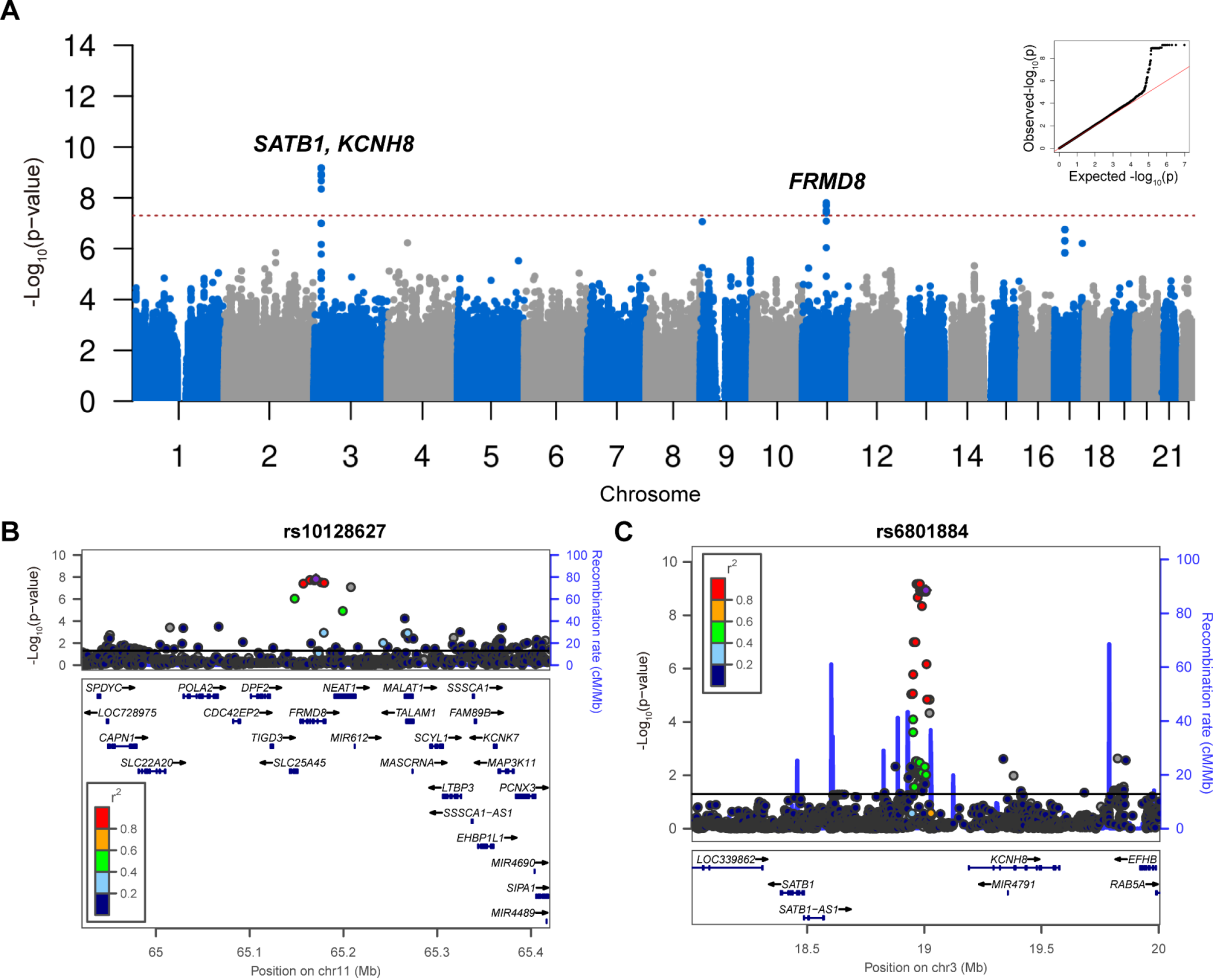
Genome-wide association analysis of AAO of ALS. (**A**) Manhattan plot showing GWAS results for AAO of ALS. (**B**) Regional plot of the association signal of rs10128627 by LocusZoom. (**C**) Regional plot of the association signal of rs6801884 by LocusZoom. Reference data of Asian population from 1,000 Genomes were used for linkage disequilibrium calculation

**Fig. 2:**
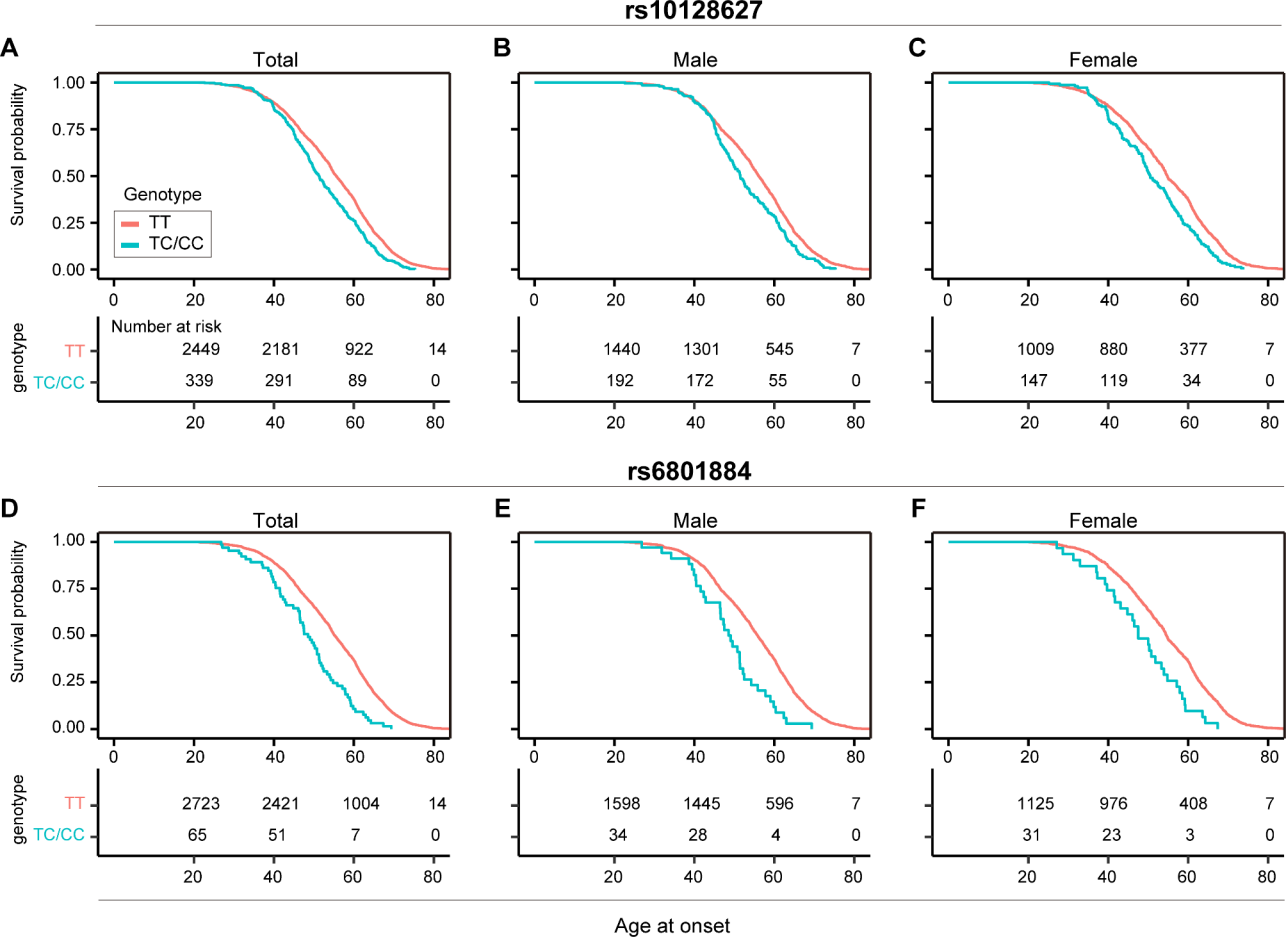
Effect of target risk loci on AAO of ALS. Kaplan-Meier survival curves of AAO of ALS (**A**) in both sexes, (**B**) in males, and (**C**) in females in relation to rs10128627. Kaplan-Meier survival curves of AAO of ALS (**D**) in both sexes, (**E**) in malesm and (**F**) in females in relation to rs6801884

In the replication stage, the locus rs10128627 was still nominally associated with AAO (P = 0.01, beta = 0.33, SE = 0.13), while the other variant rs6801884 showed no association (P = 0.54, beta = 0.15, SE = 0.25). In the pooled analysis of two stages, both variants were significantly associated with AAO of ALS, namely rs10128627 (P = 1.54E-08, beta = 0.31, SE = 0.05) and rs6801884 (P = 1.15E-09, beta = 0.77, SE = 0.13) **(**Fig. 1**)**. The variant rs10128627 resulted in earlier AAO by ~ 3.15 years, and the trend was similar in males (~ 2.86 years) and females (~ 3.25 years) (Fig. 2A-C). The variant rs6801884 resulted in earlier AAO by ~ 6.52 years, and the trend was similar in males (~ 6.59 years) and females (~ 6.32 years) (Fig. 2D-F). However, heterogeneity was detected for the locus rs6801884 using Cochran’s Q test between two stages (Cochran’s Q statistics: 88.23, Cochran’s Q P value: 3.60E-03). Therefore, further replication for this locus in additional cohort was still necessary. Gene-based association analysis showed association between *FRMD8* and AAO of ALS (P = 1.25E-06) (Supplementary Fig. 4).

### Functional annotation of the significant signals

**Fig. 3:**
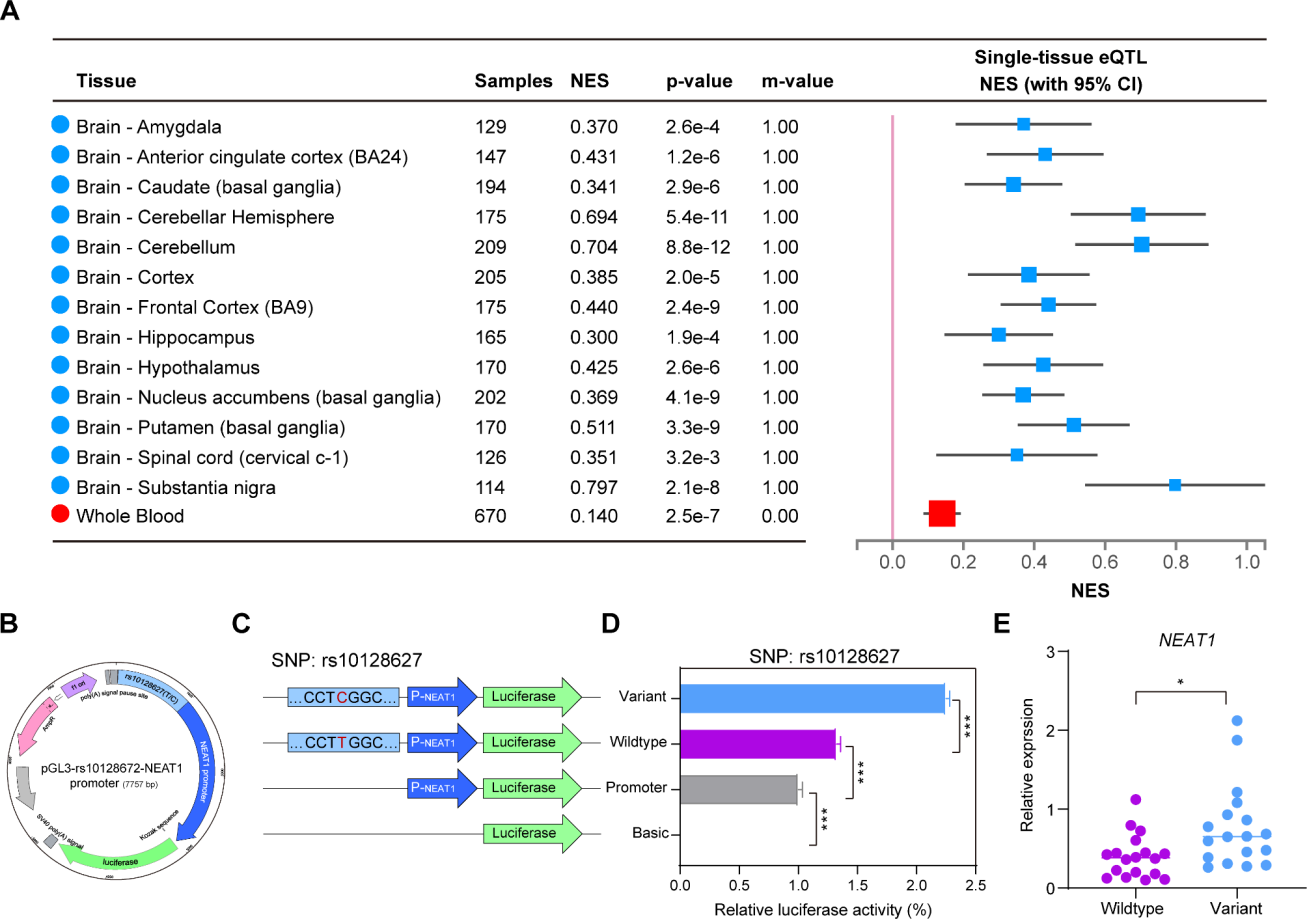
Functional analysis of lead SNP rs10128627. (**A**) The effect of rs10128627 on *NEAT1* expression in brain tissues and blood based on data from GTEx. (**B, C**) The schematic diagram shows the structure of firefly luciferase reporter plasmid. (**D**) The plasmid with the variant T > C significantly increased the ratio of firefly/Renilla luciferase reporter gene expression compared to the wild type. Data are shown as the mean ± SEM values and are the result of three independent experiments. *P < 0.05, **P < 0.01 and ***P < 0.001. NES, normalized effect size. (**E**) RT-qPCR analysis of peripheral blood mononuclear cells (PBMCs) showing higher expression of *NEAT1* in patients with variant rs10128627. P_*neat1*_ denotes the promoter of *NEAT1*.

Based on data from GTEx and QTLbase, we found that rs10128627 was a cis-eQTL for *NEAT1* in multiple brain tissues and whole blood (Table 1; Fig. 3A, Supplementary Fig. 5), while no cis-eQTL was identified for rs6801884. Given the findings from the eQTL analysis, we further performed colocalization analysis, and identified colocalization signals between locus rs10128627 and eQTLs regulating the expression of *NEAT1* in the brain and whole blood, suggesting the locus rs10128627 might be associated with the AAO of ALS by functionally modulating the expression of *NEAT1* (Supplementary Table 3). Meanwhile, a chromatin looping interaction between rs10128627 and the *NEAT1* promoter was detected using data from GeneHancer in GeneCards (GH11J065402).

**Table 1:** cis-eQTL revealing functional effects of rs10128627 in human brain tissues and whole blood

Target protein	Tissues	Effect size	P value	PMID
*Data from QTLbase*				
NEAT1	Blood	-0.34	2.78E-06	25,951,796
NEAT1	Blood	-0.36	9.33E-07	25,951,796
NEAT1	Brain	0.37	7.07E-06	25,954,001
NEAT1	Brain – Cerebellum	0.57	5.36E-07	25,954,001
LTBP3	Blood	0.14	2.43E-06	25,954,001
LTBP3	Brain - Hippocampus	-0.40	3.73E-06	32,203,495
SNX32	Brain	0.08	7.06E-06	30,545,857
*Data from GTEx*				
NEAT1	Brain - Cerebellum	0.7	8.80E-12	29,955,180
NEAT1	Brain - Cerebellar Hemisphere	0.69	5.40E-11	29,955,180
NEAT1	Brain - Frontal Cortex (BA9)	0.44	2.40E-09	29,955,180
NEAT1	Brain - Putamen (basal ganglia)	0.51	3.30E-09	29,955,180
NEAT1	Brain - Nucleus accumbens (basal ganglia)	0.37	4.10E-09	29,955,180
NEAT1	Brain - Substantia nigra	0.8	2.10E-08	29,955,180
CAPN1	Whole Blood	-0.1	9.00E-08	29,955,180
LTBP3	Whole Blood	0.11	2.10E-07	29,955,180
NEAT1	Whole Blood	0.14	2.50E-07	29,955,180
NEAT1	Brain - Anterior cingulate cortex (BA24)	0.43	1.20E-06	29,955,180
NEAT1	Brain - Hypothalamus	0.43	2.60E-06	29,955,180
NEAT1	Brain - Caudate (basal ganglia)	0.34	2.90E-06	29,955,180

To further explore the regulatory function of rs10128627 on *NEAT1* expression, we conducted a dual-luciferase reporter assay in Hela cells. We observed that the rs10128627-C risk allele significantly increased the luciferase activity relative to the rs10128627-T reference allele, indicating the T > C alteration was associated with the upregulation of *NEAT1*, consistent with the results from the cis-eQTL analysis (Fig. 3B-D). To further explore the effect of the variant rs10128627 on the expression of *NEAT1*, we assessed the RNA levels among totally 40 age- and sex-matched patients with or without rs10128627 via RT-qPCR. We found significantly higher expression of *NEAT1* in the patients with rs10128627 than those without (P = 0.012) (Fig. 3E). Taken together, the combined evidence pointed to the hypothesis that the identified locus rs10128627 might be associated with the AAO of ALS by functionally modulating the expression of *NEAT1*, which needed further exploration.

### PRS and AAO of ALS

**Fig. 4:**
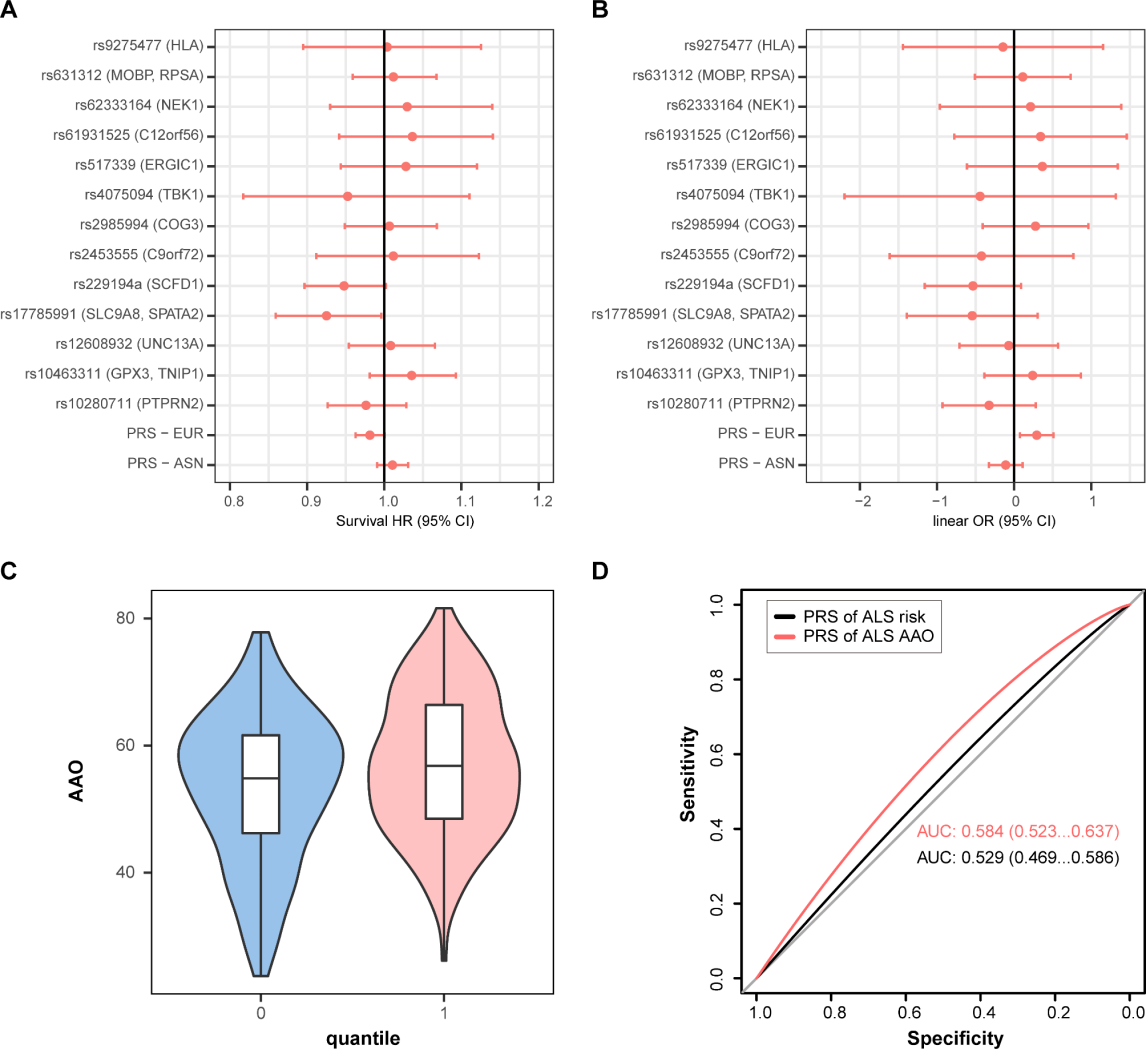
Results for target SNP and PRS analysis. (**A**) Cox proportional HRs for genome-wide significant SNPs, and PRS on age at onset (AAO) of ALS. Estimated HRs are displayed with error bars corresponding to 95% CIs. Higher effect corresponds to earlier AAO. (**B**) Effect estimates from a linear regression model of AAO of ALS. Lower effect estimates correspond to earlier AAO. Effect estimates from linear regression are displayed with error bars corresponding to 95% CIs. (**C**) Differential AAO in patients with different PRS of AAO of ALS. “0” denotes the patients in the first quartile of the PRS distribution, while “1” denotes the patients in the last quartile of the PRS distribution. (**D**) ROC curves for prediction of early-onset ALS. The black curve was plotted with PRS of risk of ALS. The red curve was plotted with PRS of AAO of ALS.

PRS based on disease risk loci identified from GWAS has been recognized as a genetic predictor for AAO of other neurodegenerative disorders like PD [29] and AD [30], while it has not been estimated for ALS. In the current study, we evaluated the association between AAO of ALS and PRS calculated from summary statistics on risk of ALS in the European and Asian populations respectively. However, we did not identify significant association (Fig. 4A, B). In the targeted analysis of risk SNPs for ALS, no variant was significantly associated with AAO of ALS. However, such results should be interpreted with caution since the significant SNPs were based on individuals of European ancestry, while these SNPs showed no association in the Asian population [14].

In addition, we calculated PRS of AAO for each individual using summary statistics of AAO of ALS from our analysis. The individuals in the first quartile of the PRS distribution had significantly earlier AAO than those in the last quartile (53.28 vs. 57.06) (Fig. 4C), suggesting PRS of AAO might provide valuable information in predicting AAO of ALS. Then we evaluated the potential utility of PRS in predicting early-onset ALS. The model using PRS from AAO of ALS could achieve a higher area under curve (AUC) of 0.59, compared with the model using PRS from risk of ALS (AUC: 0.53) (Fig. 4D).

### MR analysis

**Fig. 5:**
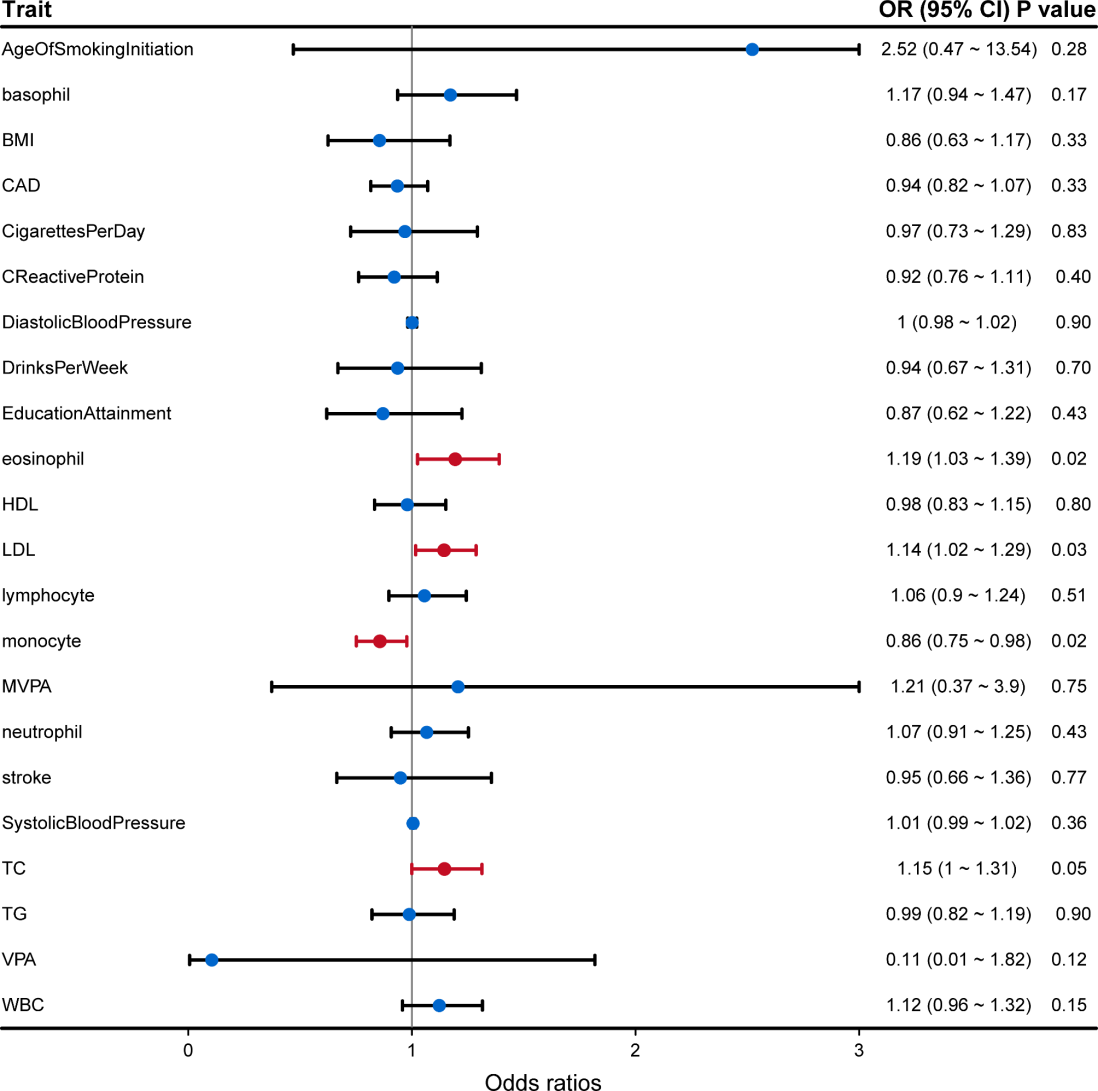
Forest plot showing results from the Mendelian randomization analysis. Estimates are per 1 standard deviation (SD) increase in the trait. Red line denotes nominal association (P < 0.05)

Previous epidemiological studies have identified a number of non-genetic risk factors implicated in the risk of ALS. However, whether they could influence AAO of ALS was still unexplored. In the current study, we studied 21 putative risk factors to infer causal association using the MR approach. Results showed that higher eosinophil, TC, and LDL were nominally associated with earlier AAO, while higher monocyte was associated later AAO of ALS (Fig. 5, Supplementary Figs. 6–9). The Cochran’s Q test did not detect heterogeneity of effects across the instrumental variables (Supplementary Table 4). The F statistics of all the instrumental variables were above 10 (ranging from 29 to 5033), indicating absence of weakness in the selected instruments. No apparent horizontal pleiotropy was observed as the intercept of MR-Egger was not significantly deviated from zero. Meanwhile, no potential instrumental outlier was detected by the MR-PRESSO analysis. The leave-one-out results suggested that the causal effect was not driven by a single instrumental variable.

## Discussion

Several GWAS have been conducted to explore the genetic factors for susceptibility of ALS, while the genetic determinants for AAO of ALS were still less explored. To fill this research gap, we performed a GWAS in 2,841 Chinese patients with ALS, and identified a novel locus rs10128627 (*FRMD8*) associated with earlier AAO of ALS by ~ 3.15 years. Colocalization and functional analyses suggested the locus rs10128627 might function by regulating the expression of *NEAT1*. Our results enhanced the current understanding of the genetic background in AAO of ALS, and provided a novel research target for the pathogenesis of ALS.

The significant locus rs10128627, which was associated with earlier AAO of ALS, was suggested to up-regulate the expression of *NEAT1* in multiple brain tissues and whole blood. Although *NEAT1* has not been identified as risk gene for ALS by GWAS, multiple functional studies on *NEAT1* suggested its essential role in neurodegenerative diseases including ALS [31]. *NEAT1* is a highly and ubiquitously expressed long non-coding RNA (lncRNA) forming a scaffold for a specific RNA granule in the nucleus and the paraspeckle, which plays an important role specifically in degenerating spinal motor neurons [32]. Previous findings have suggested that *NEAT1* might act as a scaffold of RNAs and RNA-binding proteins in the nuclei of motor neurons in ALS, thus modulating the functions of ALS-associated RNA-binding proteins during the early phase of ALS [33]. Increased paraspeckle formation has also been reported in the spinal cords of ALS patients relative to healthy individuals, suggesting paraspeckle formation might be a common hallmark of ALS patients [34]. Moreover, TDP-43 and FUS, the two major pathological proteins in ALS, both showed functional links to *NEAT1*. Using novel cell lines with the FUS gene modified by CRISPR/Cas9 and human patient fibroblasts, a previous study found that endogenous levels of mutant FUS caused accumulation of *NEAT1* isoforms and paraspeckles, and further hypothesized that perturbed structure and functionality of paraspeckles accompanied by accumulation of non-paraspeckle *NEAT1* may contribute to the disease severity in ALS-FUS [32]. Additionally, TDP-43 nuclear bodies are partially colocalized with nuclear paraspeckles, and increase of *NEAT1* promotes TDP-43 liquid-liquid phase separation (LLPS) in vitro [35]. In addition, *NEAT1* is highly enriched in neurons of the anterior horn of the spinal cord and cortical tissues of ALS patients [34, 36]. These multiple lines of evidence suggested the essential role of *NEAT1* in the pathogenesis of ALS. In the current study, we illuminated the role of *NEAT1* in the pathology of AAO of ALS from genetic and functional perspectives. The significant locus rs10128627 was suggested to up-regulate the expression of *NEAT1*, while CRISPR-assisted up-regulation of endogenous *NEAT1* has been shown to cause neurotoxicity [37]. In our study, patients with the minor allele of rs10128627 showed earlier AAO by ~ 3.15 years. Cis-eQTL and enhancer analysis suggested the variant rs10128627 might upregulate the expression of *NEAT1*, while dual-luciferase reporter assay and RT-qPCR experiments further reinforced such hypothesis. Therefore, upregulated expression of *NEAT1* might expedite the development of ALS and thus lead to earlier AAO, though further exploration was still necessary to investigate how this variant was involved in the pathogenesis of the disease. Notably, this variant was nominally associated with risk of ALS in two previous GWAS on ALS susceptibility (P = 0.027; beta = 0.04 and P = 0.02; beta = 0.04 respectively) [15, 38], suggesting this variant might increase the risk of ALS as well, consistent with our results that this variant was associated with earlier AAO. These results suggested potential shared pathogenesis between AAO and risk of ALS. However, another subsequent study showed insignificant association for this variant (P = 0.511), though the effect direction was the same (beta = 0.01) [39]. In addition, no significant association was identified for this variant in previous GWAS on AAO of ALS in the European population [8]. Notably, The MAF of the variant rs10128627 differs between different populations, ranging from 0.06 in East Asians, to 0.16 in Europeans and 0.26 in Latino/Admixed American based on data from gnomAD v3.1.2 (Supplementary Fig. 10), suggesting potential different effect of this variant in different populations. Therefore, further exploration in additional cohorts was warranted to better understand the association between this variant and disease risk and progression.

Another intergenic locus rs6801884 was significantly associated with AAO of ALS in the discovery stage. We tried to locate the driver gene for the significant locus using SnpEff and existent cis-eQTL evidence in available eQTL resources including GTEx, eQTLGen, Braineac, Brain xQTL Serve, and PsychEncode, but failed to map rs6801884 to a functional gene. Regulatory functions searched in RegulomeDB and HaploReg did not show clear associations either. Around the locus rs6801884 were two protein-coding genes *KCNH8* and *SATB1*. Functionally, *KCNH8* encodes a voltage-gated potassium channel that is primarily expressed in the nervous system, and plays a role in transmission pathways across chemical synapses and potassium channels, which is closely involved in the pathogenesis of ALS [40]. It has been suggested that the fasciculations in ALS are caused by an imbalance between functional sodium and potassium channels, and this ion channel dysfunction could be responsible for the motor neuron degeneration [41]. The main functions of *KCNH8* include regulating neurotransmitter release and neuronal excitability. Previous study has demonstrated motor neuron hyperexcitability for ALS and its sensitivity to Kv7 agonists across iPSC lines, patients, and genotypic etiologies, and differential expression of voltage-gated potassium channels including *KCNH8* was observed in ALS motor neurons [42]. These evidence suggested the potential role of *KCNH8* in the pathogenesis of ALS through regulating potassium channel abnormalities. *SATB1* is a ubiquitously expressed chromatin organizing factor, and mediates the transcriptional response of midbrain dopamine neurons to toxic insult [43]. Previous GWAS have identified *SATB1* as a risk gene for PD, another common neurodegenerative disorder [44, 45], while no prominent reports about *SATB1* in ALS were found. One lncRNA (SATB1-AS1) and one miRNA (MIR4791) were in the vicinity of this variant, but the functions of the two RNAs were still poorly studied, and we did not find evidence supporting their potential role in the pathogenesis of ALS. Notably, no significant association was identified for the variant rs6801884 in the replication phase, and Cochran’s Q test detected heterogeneity in the meta-analysis of discovery and replication stages. Failure of replication might be due to limited sample size since the MAF of this variant rs6801884 in East Asians is low (MAF = 0.01) (Supplementary Fig. 11). There is a high risk that the results might be susceptible to bias from the small sample size and the observed effect was due to chance. Therefore, further replication was necessary to verify the association between this locus and AAO of ALS.

Common genetic variants associated with ALS risk have a small but consistent effect on disease etiology, and might combine to expedite the onset of the disease. In the current study, we did not identify association between PRS and AAO of ALS, consistent with a recent study in the European population [14]. However, the results should be interpreted with caution due to the population stratification across populations and limited sample size of the Asian GWAS. Further exploration based on summary statistics from larger GWAS was still warranted. In addition, we demonstrated that PRS of AAO could aid in the prediction of early-onset ALS, suggesting potential to apply PRS of AAO in clinical trials to identify patients susceptible to early-onset ALS.

Previous observational studies have shown conflicting evidence regarding different risk factors in the risk of ALS. The unavoidable confounding factors and reverse causality also challenged the interpretation of these results. Using the MR approach, we demonstrated that higher TC and LDL were causally associated with earlier AAO of ALS. Correspondingly, previous studies have shown that higher TC and LDL might increase risk of ALS [14, 46, 47], suggesting the two lipids might play a harmful role in the pathogenesis of ALS. Neuroinflammation also plays an important role in the pathophysiology of ALS. Previous study has shown that changes in peripheral inflammatory markers contribute to the pathologic features of ALS [48]. In the current study, we demonstrated that higher eosinophil levels were causally associated with earlier AAO, while higher monocyte levels might delay the onset age. Similarly, infiltration of peripheral monocytes in the central nervous system was suggested to improve motor neuron survival in a genetic ALS mouse model [49], suggesting the potential protective role of monocytes in the disease. Meanwhile, eosinophil-derived neurotoxin level was significantly increased in the serum of patients with ALS as compared with healthy controls, suggesting the potentially harmful role of eosinophil in the pathophysiology of ALS [50]. However, the results should be interpreted with caution since only nominal association was identified. Meanwhile, the instrumental variables were obtained from GWAS in the European population, which might bring some bias. Therefore, further replication was still warranted.

## Conclusions

We performed a GWAS on AAO of ALS in a large Chinese ALS cohort and identified a novel locus which might lead to earlier AAO by regulating the expression of *NEAT1*. Our findings supplement the current knowledge on the genetic architecture for AAO of ALS, and provide a novel target for further research on the pathogenesis of ALS and potential therapeutic options to delay the disease onset.

### Electronic supplementary material

Below is the link to the electronic supplementary material.

Supplementary Material 1. **Additional file 1**: **Supplementary Figure 1**. Age at onset distribution in the patients with ALS. **Supplementary Figure 2**. Effect of gender on age at onset of ALS. **Supplementary Figure 3**. Principal component analysis plot of populations from 1000 Genomes Project and the ALS patients in our cohort. **Supplementary Figure 4**. Results from genome-wide gene-based association analysis. **Supplementary Figure 5**. QTLbase variant-level query results for rs10128627. **Supplementary Figure 6**. Mendelian randomization analysis results for eosinophil on age at onset of ALS. **Supplementary Figure 7**. Mendelian randomization analysis results for monocyte on age at onset of ALS. **Supplementary Figure 8**. Mendelian randomization analysis results for LDL cholesterol on age at onset of ALS. **Supplementary Figure 9**. Mendelian randomization analysis results for total cholesterol on age at onset of ALS. **Supplementary Figure 10**. Minor allele frequency of variant rs10128627 in different populations. **Supplementary Figure 11**. Minor allele frequency of variant rs6801884 in different populations.

Supplementary Material 2. **Additional file 2**: **Supplementary Table 1**. Summary data of GWAS used in the Mendelian randomization analysis. **Supplementary Table 2**. Demographics of the study subjects. **Supplementary Table 3**. Candidate risk genes identified by colocalization analysis. **Supplementary Table 4**. Results from heterogeneity and horizontal pleiotropy analysis.

Supplementary Material 3. **Additional file 3**: The sequence of the NEAT1 promoter region.

## Data Availability

Summary statistics of the risk of ALS can be found in the original publication. Summary statistics of the risk factors for ALS in Mendelian randomization can be found in the original publication.

## Abbreviations

ALS: amyotrophic lateral sclerosis
GWAS: genome-wide association study
SNP: single nucleotide polymorphisms
MAF: minor allele frequency
PCA: principal component analysis
PRS: polygenic risk score
AUC: area under curve
ROC: receiver operating characteristic
eQTL: expression quantitative trait loci
LS: linkage disequilibrium
MR: Mendelian randomization
IVW: inverse variance weighted
RT-qPCR: reverse transcription-quantitative PCR
TC: total cholesterol
LDL: low-density lipoprotein
HDL: high-density lipoprotein
