# Supplementary material for: Genome-wide analyses identify NEAT1 as genetic modifier of age at onset of amyotrophic lateral sclerosis
Source: Mol Neurodegener. 2023 Oct 23;18:77. doi: 10.1186/s13024-023-00669-6 (PMC10594666; doi:10.1186/s13024-023-00669-6)
Supplement: Supplementary file 1 — Supplementary Material 1. Additional file 1: Supplementary Figure 1. Age at onset distribution in the patients with ALS. Supplementary Figure 2. Effect of gender on age at onset of ALS. Supplementary Figure 3. Principal component analysis plot of populations from 1000 Genomes Project and the ALS patients in our cohort. Supplementary Figure 4. Results from genome-wide gene-based association analysis. Supplementary Figure 5. QTLbase variant-level query results for rs10128627. Supplementary Figure 6. Mendelian randomization analysis results for eosinophil on age at onset of ALS. Supplementary Figure 7. Mendelian randomization analysis results for monocyte on age at onset of ALS. Supplementary Figure 8. Mendelian randomization analysis results for LDL cholesterol on age at onset of ALS. Supplementary Figure 9. Mendelian randomization analysis results for total cholesterol on age at onset of ALS. Supplementary Figure 10. Minor allele frequency of variant rs10128627 in different populations. Supplementary Figure 11. Minor allele frequency of variant rs6801884 in different populations. [file 13024_2023_669_MOESM1_ESM.docx]

**Supplementary Figure 1. Age at onset distribution in the patients with ALS.**
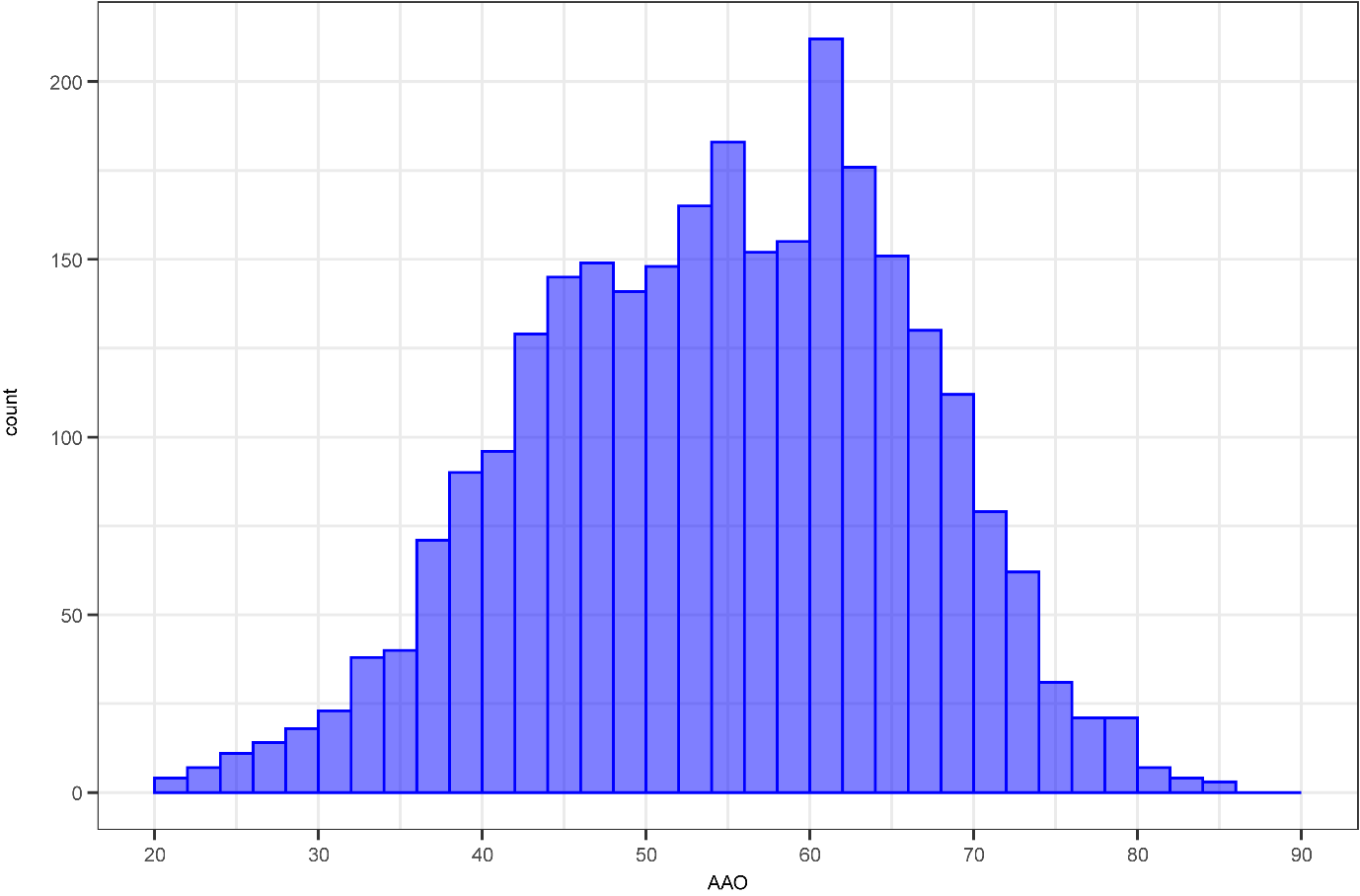


**Supplementary Figure 2. Effect of gender on age at onset of ALS.**


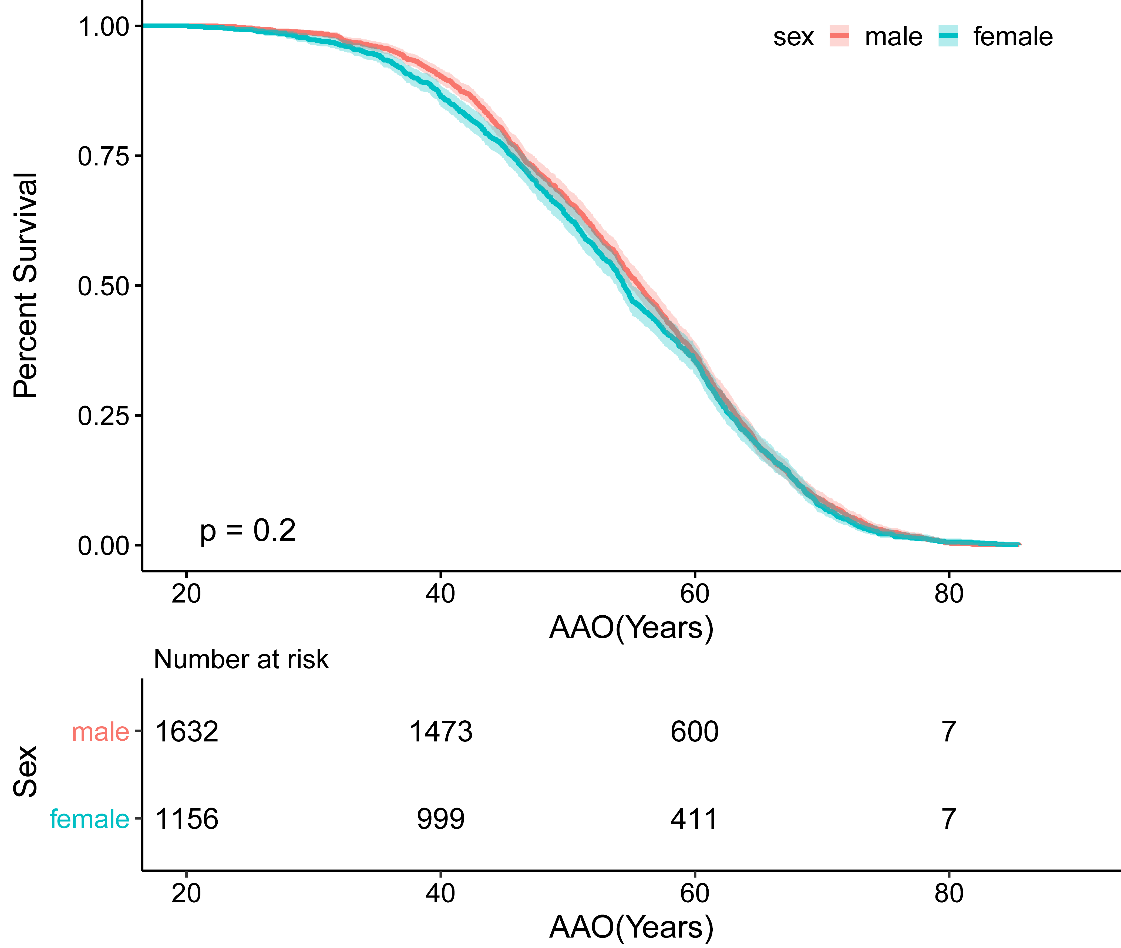


**Supplementary Figure 3. Principal component analysis plot of populations from 1000 Genomes Project and the ALS patients in our cohort.**


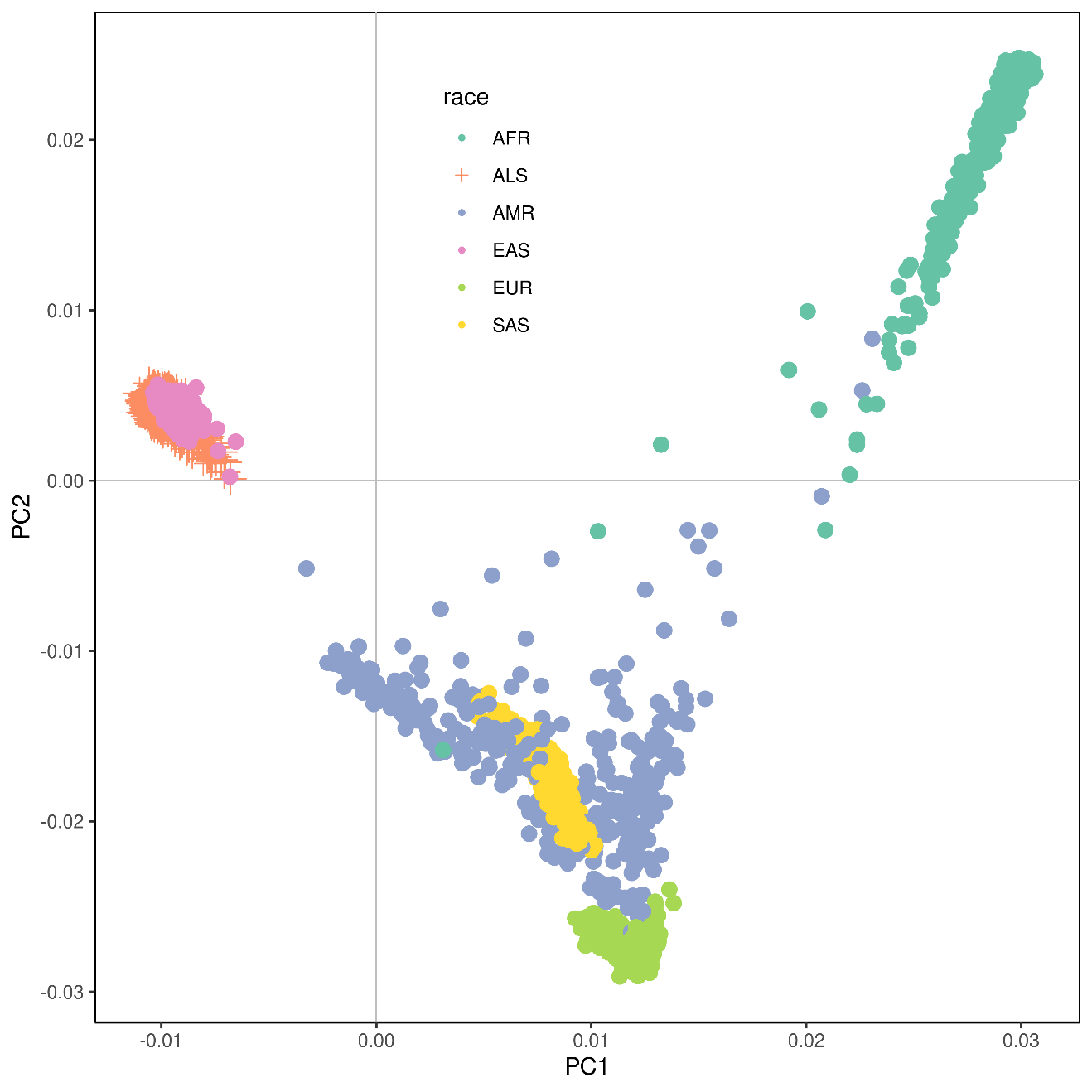


Individuals from 1000 Genomes Project and our dataset were analyzed. AFR, African; ALS, Chinese ALS patients in our cohort; AMR, American; EAS: East Asian, EUR: European; SAS, South Asian; PC, principal component.

**Supplementary Figure 4. Results from genome-wide gene-based association analysis.**


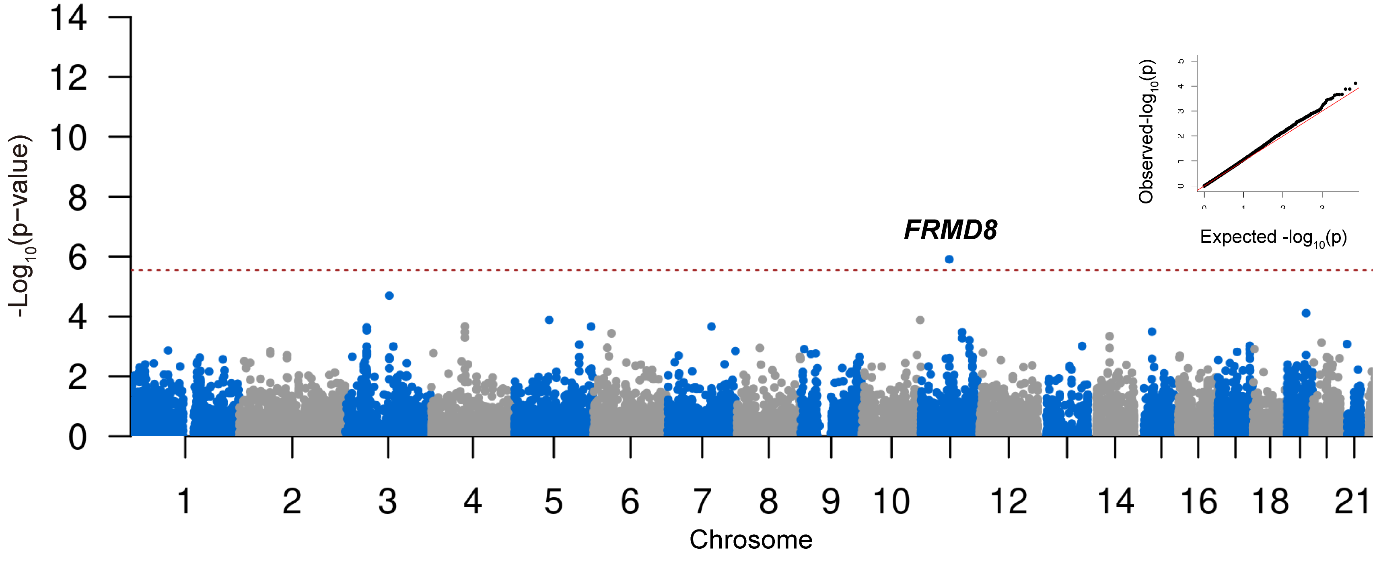


**Supplementary Figure 5. A heat map plot of associated QTLs across traits and tissues for the variant rs10128627.**


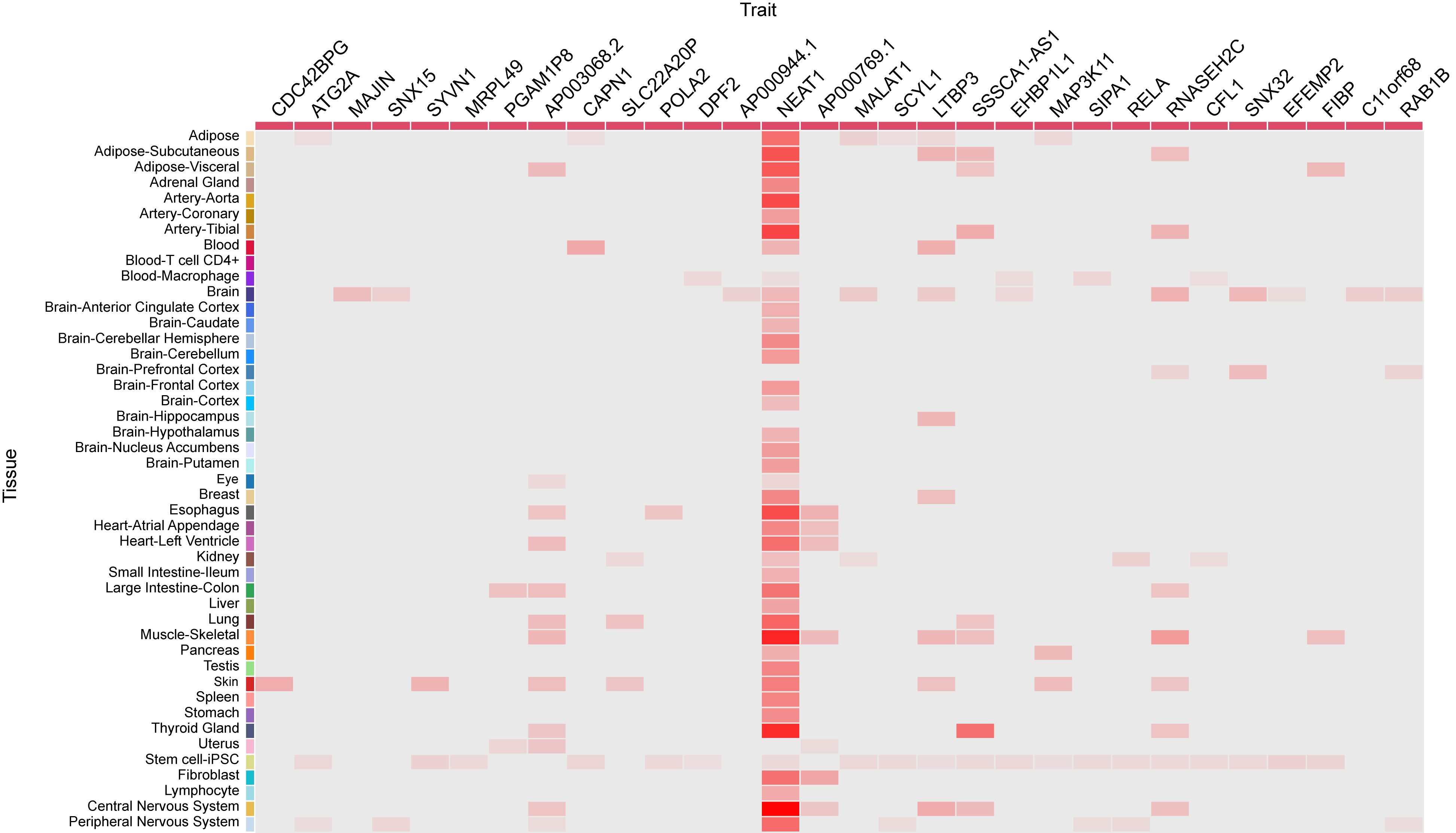


Results were queried in QTLbase. Associations with P<0.01 were shown.

**Supplementary Figure 6. Mendelian randomization analysis results for** **eosinophil on age at onset of ALS.**


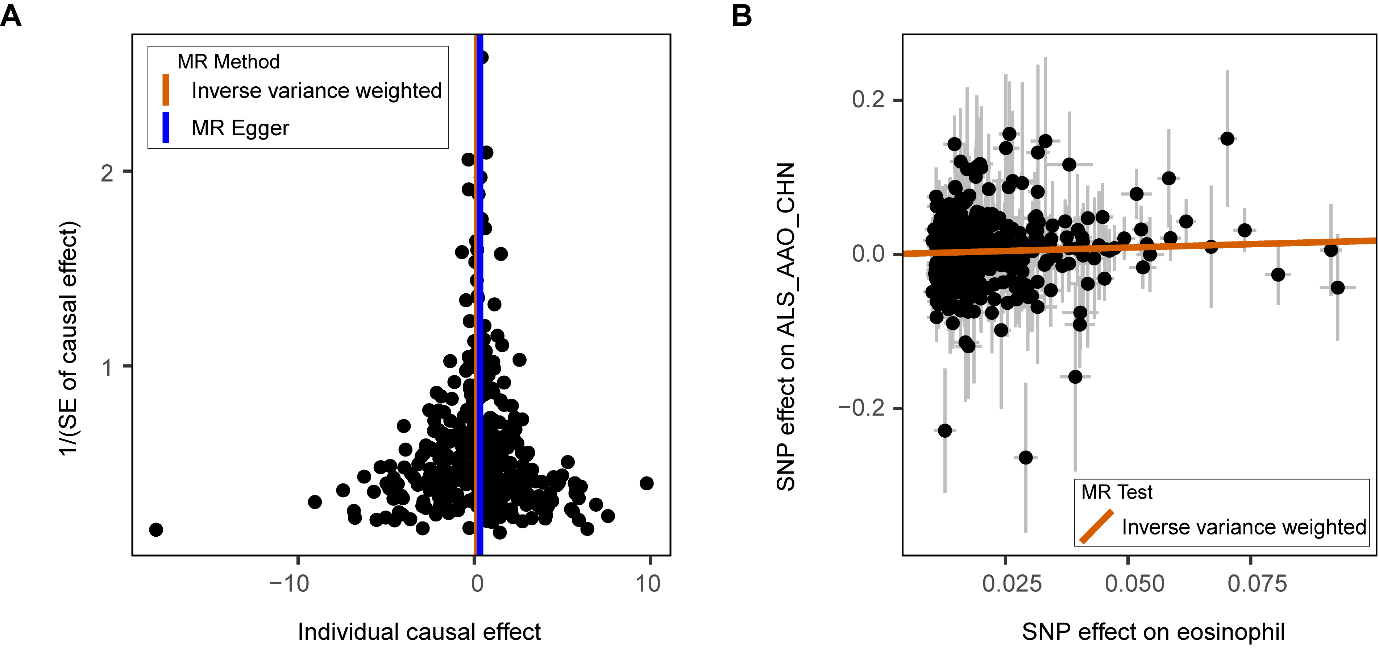


(**A**) Funnel plot of single-SNP effect estimates and corresponding inverse standard errors. (**B**) Scatter plot of genetic associations with eosinophil (horizontal lines) against genetic associations with age at onset of ALS (vertical lines). Error bars for genetic associations are 95% confidence intervals. The slopes of each line in the scatter plot represent the causal association for each method.

**Supplementary Figure 7. Mendelian randomization analysis results for monocyte on age at onset of ALS.**


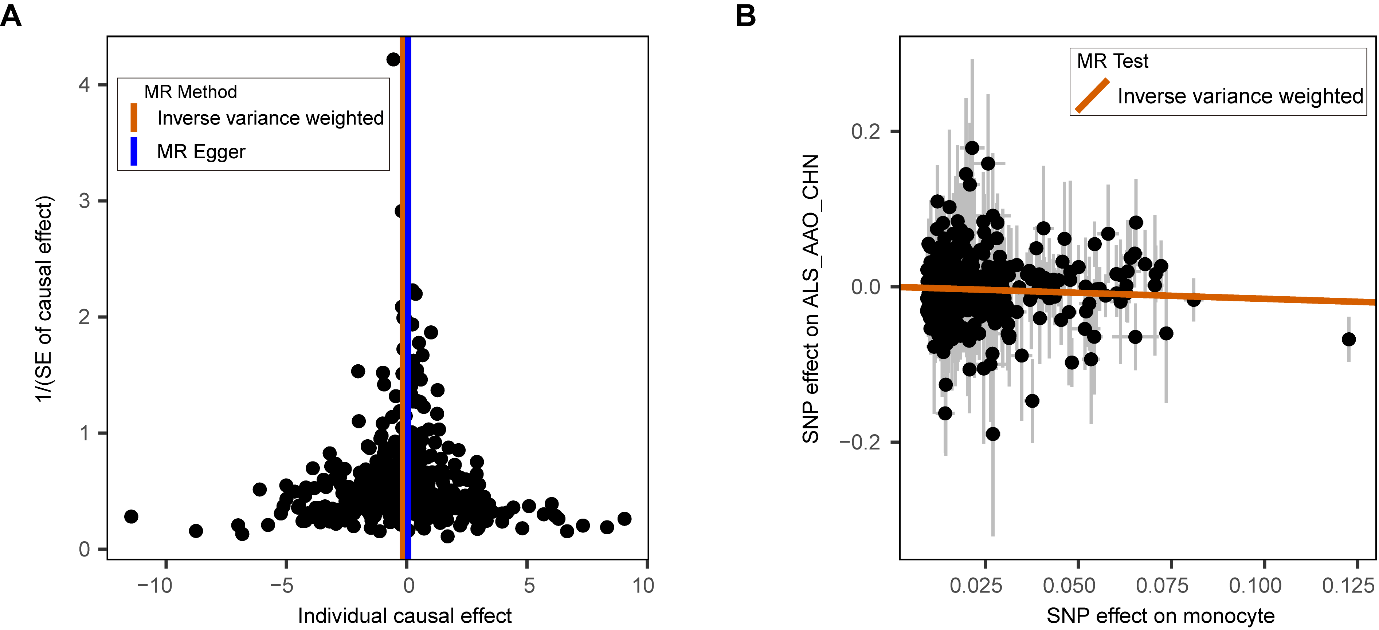


**Supplementary Figure 8. Mendelian randomization analysis results for LDL cholesterol on age at onset of ALS.**


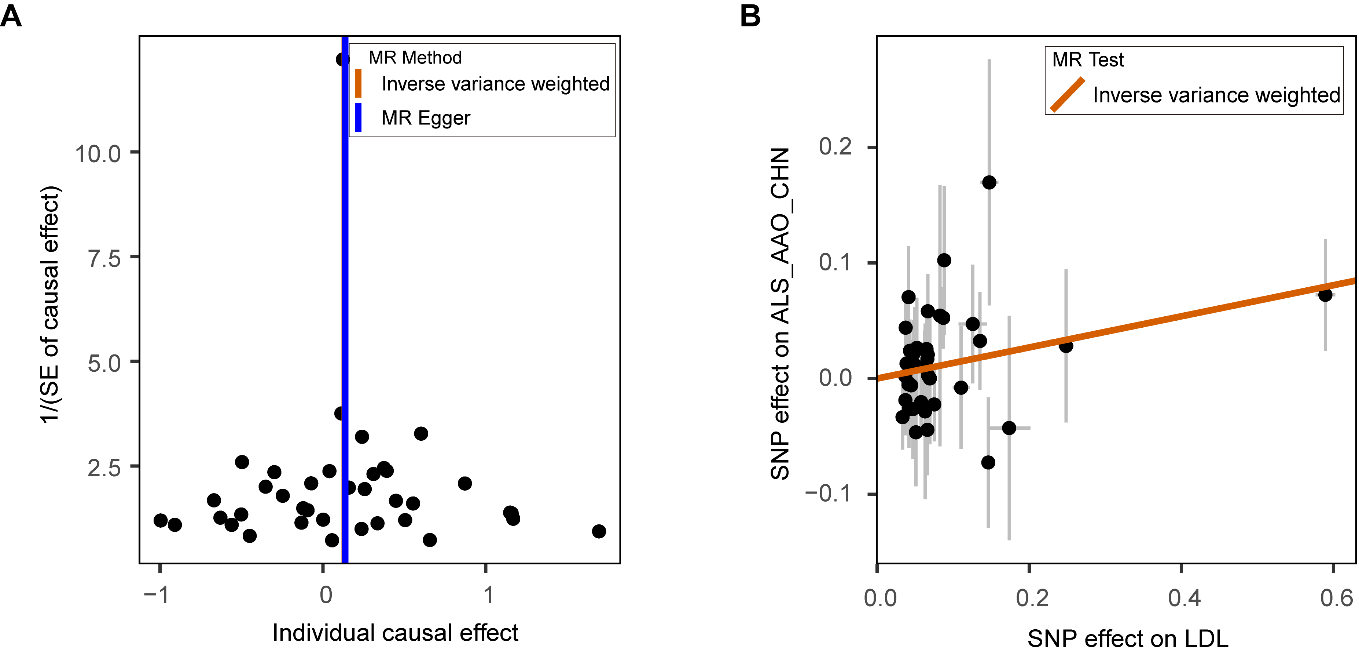


**Supplementary Figure 9. Mendelian randomization analysis results for total cholesterol on age at onset of ALS.**


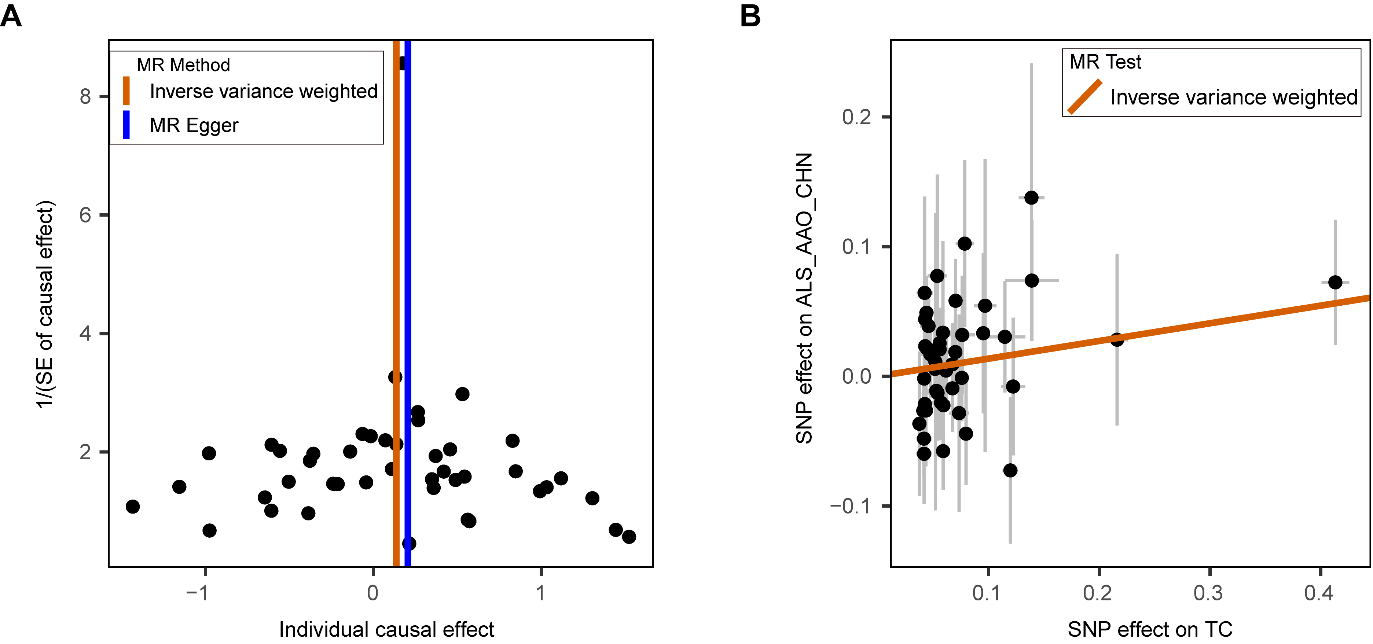


**Supplementary Figure 10. Minor allele frequency of variant rs10128627 in different populations.**


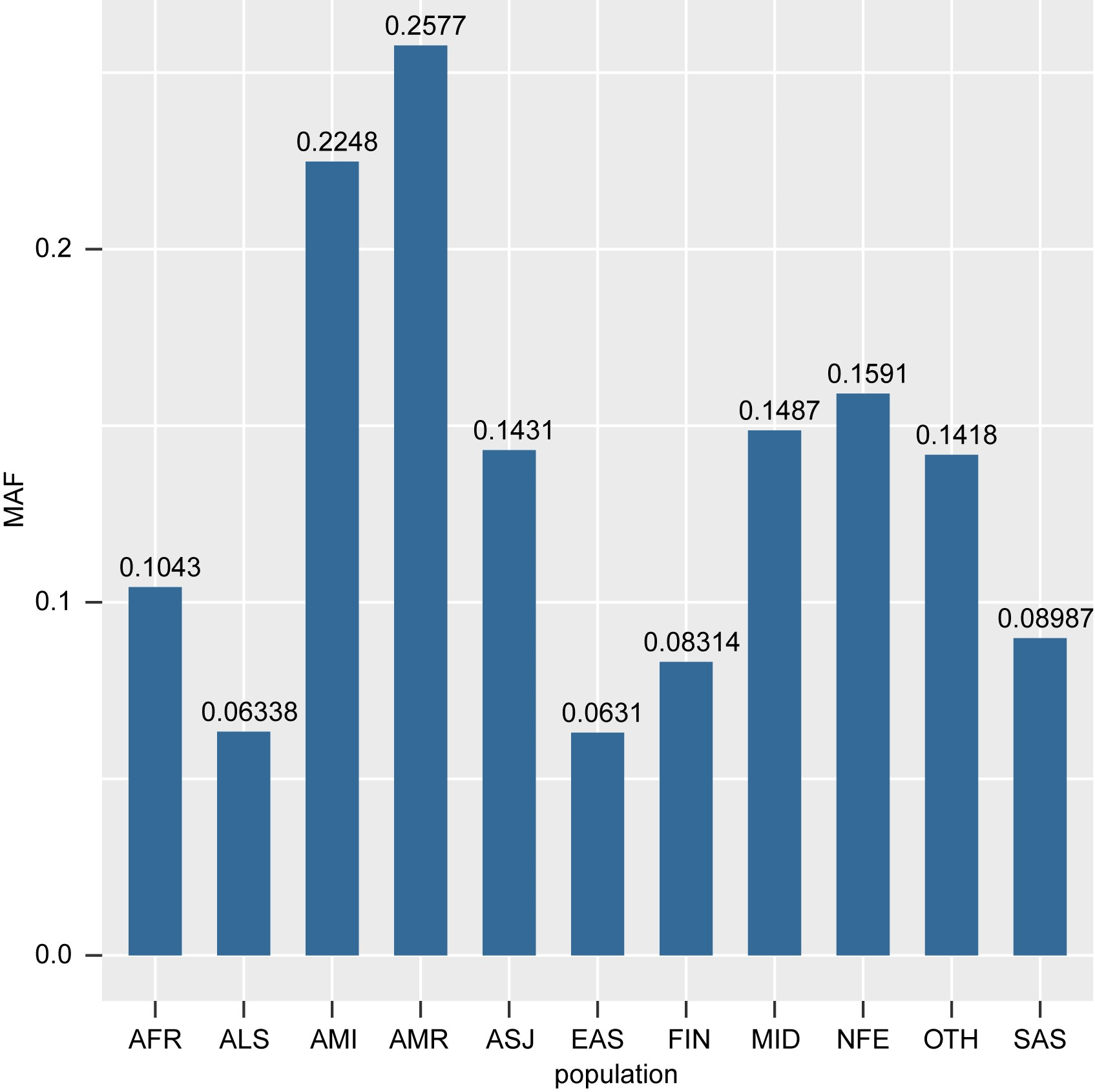


MAF, minor allele frequency; AFR, African/African American; ALS, patients with ALS in the current Chinese cohort; AMI, Amish; AMR, Latino/Admixed American; ASJ, Ashkenazi Jewish; EAS: East Asian; FIN, Finnish; NFE, Non-Finnish European; MID, Middle Eastern; SAS, South Asian; OTH: Other (population not assigned). MAF information was obtained from gnomAD v 3.1.2.

**Supplementary Figure 11. Minor allele frequency of variant rs6801884 in different populations.**


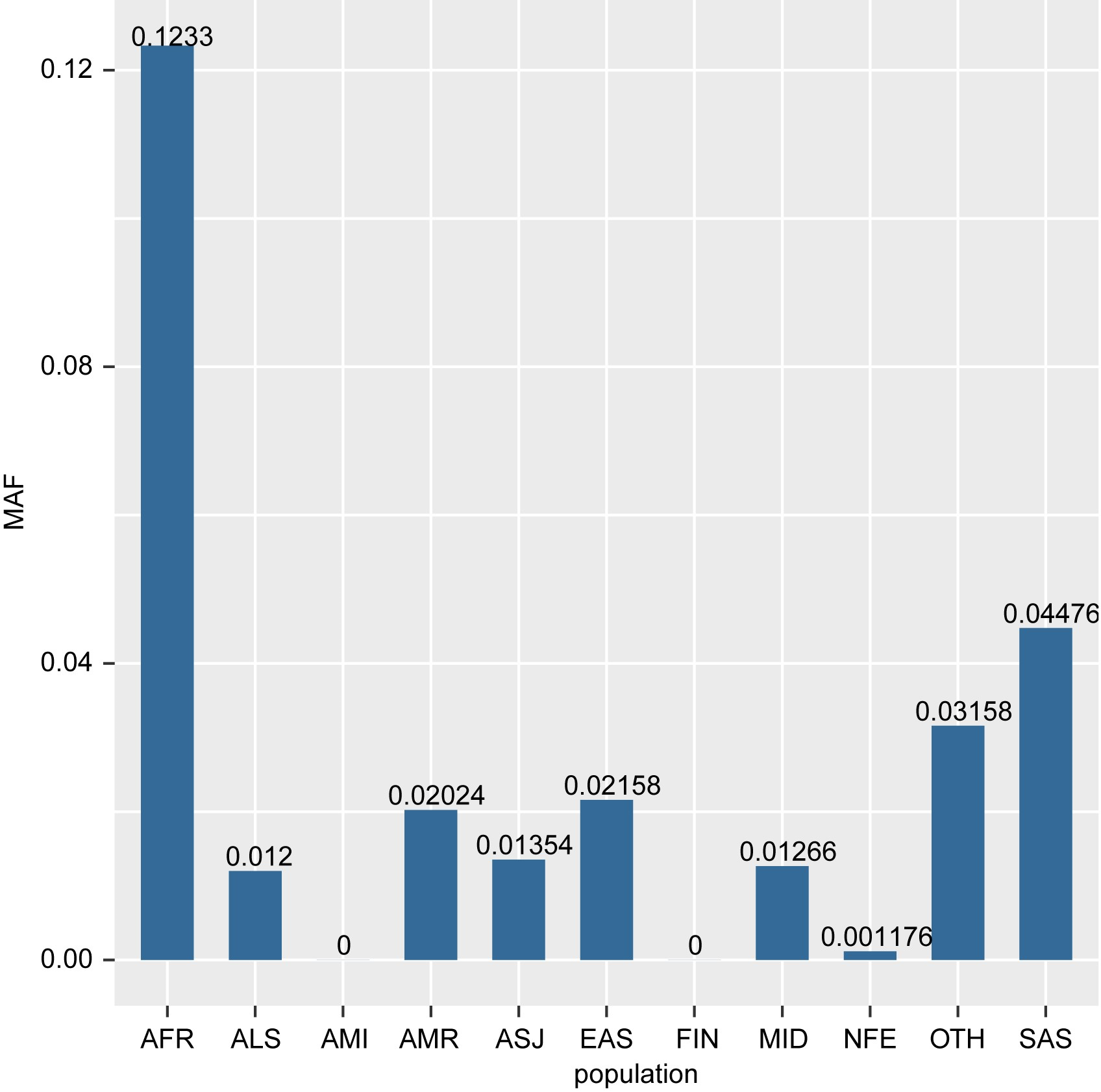


MAF, minor allele frequency; AFR, African/African American; ALS, patients with ALS in the current Chinese cohort; AMI, Amish; AMR, Latino/Admixed American; ASJ, Ashkenazi Jewish; EAS: East Asian; FIN, Finnish; NFE, Non-Finnish European; MID, Middle Eastern; SAS, South Asian; OTH: Other (population not assigned). MAF information was obtained from gnomAD v 3.1.2.
