# Supplementary material for: Genome-wide analyses identify NEAT1 as genetic modifier of age at onset of amyotrophic lateral sclerosis
Source: Mol Neurodegener. 2023 Oct 23;18:77. doi: 10.1186/s13024-023-00669-6 (PMC10594666; doi:10.1186/s13024-023-00669-6)
Supplement: Supplementary file 2 — Supplementary Material 2. Additional file 2: Supplementary Table 1. Summary data of GWAS used in the Mendelian randomization analysis. Supplementary Table 2. Demographics of the study subjects. Supplementary Table 3. Candidate risk genes identified by colocalization analysis. Supplementary Table 4. Results from heterogeneity and horizontal pleiotropy analysis. [file 13024_2023_669_MOESM2_ESM.docx]

**Supplementary Table 1. Summary data of GWAS used in the Mendelian randomization analysis.**

| trait | Cases | Controls | Ethnics | PMID |
| --- | --- | --- | --- | --- |
| body mass index | 339,224 | - | EUR | 25673413 |
| years of schooling | 1,131,881 | - | EUR | 30038396 |
| alcoholic drinks per week | 941,280 | - | EUR | 30643251 |
| age of smoking initiation | 1,232,091 | - | EUR | 30643251 |
| cigarettes per day | 337,334 | - | EUR | 30643251 |
| moderate physical activity | 377,234 | - | EUR | 29899525 |
| vigorous activity | 98,060 | - | EUR | 29899525 |
| coronary artery disease | 113,937 | 339,115 | EUR | 28714975 |
| diastolic blood pressure | 757,601 | - | EUR | 30224653 |
| systolic blood pressure | 757,601 | - | EUR | 30224653 |
| basophil | 503,085 | - | EUR | 32888494 |
| eosinophil | 503,085 | - | EUR | 32888494 |
| lymphocyte | 503,085 | - | EUR | 32888494 |
| monocyte | 503,085 | - | EUR | 32888494 |
| neutrophil | 503,085 | - | EUR | 32888494 |
| white blood cell counts | 503,085 | - | EUR | 32888494 |
| C-reactive protein | 204,402 | - | EUR | 30388399 |
| HDL cholesterol | 62,166 | - | EUR | 25961943 |
| LDL cholesterol | 62,166 | - | EUR | 25961943 |
| total cholesterol | 62,166 | - | EUR | 25961943 |
| triglyceride levels | 62,166 | - | EUR | 25961943 |

EUR, European; GWAS, genome-wide association study; PMID, PubMed ID.

**Supplementary Table 2. Demographics of the study subjects.**

| **Group** | **No.** | **AAO, years (SD)** | **Male/Female ratio** | **AAO in males** | **AAO in females** |
| --- | --- | --- | --- | --- | --- |
| discovery | 2230 | 54.56 (11.56) | 1.40 (1301/929) | 54.82 (11.29) | 54.19 (11.93) |
| replication | 558 | 54.69 (11.33) | 1.46 (331/227) | 55.68 (11.12) | 53.25 (11.52) |
| both stages | 2788 | 54.59 (11.52) | 1.41 (1632/1156) | 55.00 (11.26) | 54.00 (11.85) |

No., number of individuals; AAO, age at onset; SD, standard deviation.

**Supplementary Table 3. Candidate risk genes identified by colocalization analysis.**

| **Tissue** | **Gene** | **PP.H0.abf** | **PP.H1.abf** | **PP.H2.abf** | **PP.H3.abf** | **PP.H4.abf** |
| --- | --- | --- | --- | --- | --- | --- |
| Brain_Anterior_cingulate_cortex_BA24 | *NEAT1* | 2.75E-05 | 5.32E-03 | 2.82E-05 | 4.47E-03 | 0.99 |
| Brain_Caudate_basal_ganglia | *NEAT1* | 4.76E-05 | 9.22E-03 | 2.87E-05 | 4.58E-03 | 0.99 |
| Brain_Cerebellar_Hemisphere | *NEAT1* | 4.86E-08 | 9.41E-06 | 2.91E-05 | 4.63E-03 | 0.995 |
| Brain_Cerebellum | *SLC22A20P* | 6.65E-03 | 1.12E-01 | 5.26E-05 | 0 | 0.88 |
| Brain_Cerebellum | *NEAT1* | 1.48E-08 | 2.86E-06 | 2.96E-05 | 4.73E-03 | 0.995 |
| Brain_Cortex | *NEAT1* | 1.45E-04 | 2.82E-02 | 3.13E-05 | 5.10E-03 | 0.97 |
| Brain_Frontal_Cortex_BA9 | *NEAT1* | 6.05E-07 | 1.17E-04 | 2.68E-05 | 4.20E-03 | 0.996 |
| Brain_Hypothalamus | *NEAT1* | 5.24E-05 | 1.02E-02 | 2.82E-05 | 4.47E-03 | 0.99 |
| Brain_Nucleus_accumbens_basal_ganglia | *NEAT1* | 5.32E-07 | 1.03E-04 | 3.17E-05 | 5.14E-03 | 0.99 |
| Brain_Putamen_basal_ganglia | *NEAT1* | 6.34E-07 | 1.23E-04 | 2.91E-05 | 4.64E-03 | 0.995 |
| Brain_Substantia_nigra | *NEAT1* | 2.25E-06 | 4.37E-04 | 2.94E-05 | 4.70E-03 | 0.99 |
| Whole_Blood | *CPAN1* | 4.91E-06 | 9.52E-04 | 2.89E-05 | 4.61E-03 | 0.99 |
| Whole_Blood | *NEAT1* | 6.58E-06 | 1.27E-03 | 2.94E-05 | 4.71E-03 | 0.99 |
| Whole_Blood | *LTBP3* | 8.51E-06 | 1.65E-03 | 3.39E-05 | 5.57E-03 | 0.99 |
| Whole_Blood | *RP11-770G2.2* | 3.04E-03 | 1.23E-01 | 4.45E-05 | 9.34E-04 | 0.87 |
| Whole_Blood | *CMB9-22P13.1* | 2.59E-03 | 1.02E-01 | 2.27E-05 | 0 | 0.90 |

**Supplementary Table 4. Results from heterogeneity and horizontal pleiotropy analysis.**

| exposure trait | Heterogeneity | | | Horizontal pleiotropy | | | MR-PRESSO  P value |  |
| --- | --- | --- | --- | --- | --- | --- | --- | --- |
|  | IVW Q | Q df | P value | Egger intercept | SE | P value |  |  |
| body mass index | 81.05 | 68 | 0.133 | 2.1E-02 | 0.011 | 0.052 | 0.128 |  |
| years of schooling | 300.30 | 263 | 0.057 | -6.5E-03 | 0.009 | 0.486 | 0.054 |  |
| alcoholic drinks per week | 34.71 | 25 | 0.094 | 6.3E-04 | 0.010 | 0.949 | 0.354 |  |
| age of smoking initiation | 2.12 | 3 | 0.547 | 3.7E-02 | 0.162 | 0.839 | 0.579 |  |
| cigarettes per day | 15.05 | 20 | 0.773 | 8.2E-03 | 0.018 | 0.656 | 0.783 |  |
| moderate physical activity | | 14.33 | 15 | 0.501 | -2.3E-02 | 0.040 | 0.568 | 0.496 |
| vigorous activity | 4.14 | 6 | 0.657 | 1.2E-01 | 0.132 | 0.417 | 0.65 |  |
| coronary artery disease | 29.80 | 37 | 0.794 | 3.0E-03 | 0.015 | 0.846 | 0.802 |  |
| diastolic blood pressure | 412.57 | 376 | 0.094 | -6.1E-03 | 0.005 | 0.180 | 0.093 |  |
| systolic blood pressure | 423.53 | 384 | 0.080 | 4.0E-03 | 0.004 | 0.348 | 0.088 |  |
| basophil | 168.06 | 182 | 0.763 | 5.1E-03 | 0.005 | 0.284 | 0.761 |  |
| eosinophil | 396.97 | 383 | 0.301 | -4.0E-03 | 0.004 | 0.291 | 0.282 |  |
| lymphocyte | 403.00 | 430 | 0.821 | -1.6E-03 | 0.004 | 0.672 | 0.834 |  |
| monocyte | 372.03 | 424 | 0.967 | -6.1E-03 | 0.003 | 0.430 | 0.971 |  |
| neutrophil | 365.28 | 362 | 0.442 | 5.6E-03 | 0.004 | 0.138 | 0.439 |  |
| white blood cell counts | 473.53 | 456 | 0.276 | 2.5E-03 | 0.003 | 0.469 | 0.273 |  |
| C-reactive protein | 38.02 | 48 | 0.848 | 3.9E-03 | 0.008 | 0.637 | 0.849 |  |
| HDL cholesterol | 34.82 | 33 | 0.382 | 1.9E-02 | 0.011 | 0.108 | 0.414 |  |
| LDL cholesterol | 27.86 | 37 | 0.861 | -2.8E-04 | 0.008 | 0.974 | 0.889 |  |
| total cholesterol | 38.04 | 43 | 0.686 | -6.8E-03 | 0.009 | 0.470 | 0.711 |  |
| triglyceride levels | 24.27 | 26 | 0.561 | -7.8E-03 | 0.015 | 0.596 | 0.545 |  |

IVW, Inverse variance weighted; Q, Cochran’s Q test estimate; df, Cochran’s Q test degrees of freedom; SE, standard error.
