# Supplementary material for: Genome-wide analyses identify NEAT1 as genetic modifier of age at onset of amyotrophic lateral sclerosis
Source: Mol Neurodegener. 2023 Oct 23;18:77. doi: 10.1186/s13024-023-00669-6 (PMC10594666; doi:10.1186/s13024-023-00669-6)
Supplement: Supplementary file 3 — Supplementary Material 3. Additional file 3: The sequence of the NEAT1 promoter region. [file 13024_2023_669_MOESM3_ESM.docx]

**The sequence of the *NEAT1* promoter region.**

TCTTGCTGTGTTTCCCAGGCTGGTCTCGAACTCCTGGCCTCTAGGCAATCCTCCCACCATGGCCTTCAAAAGCGAATGGATCCCACTCTTTTTTGAAAAATAAAATCTACCATAGTTTGTTTCTGGGATTTGGGAATGATGTGTGTGAATTAGAGAGATTTCGAGACCTGAAATAGAGAAAAGCAAGTTAAGACATCAACTGGGGCCCAGAAACAGCACTACAGCGGGGACGACCCACCTCTCAGATGCCTGGTGAGATAAAGAAAGGTCTGGCTGACTCCACTGCCCAGTACTGTGAAGCAGCAGTGAAGACTCCTCCATTGGTTTCATAAGCTGCCCAGCTTTCTAGAACATTTTGACAGACAATTCCCTCTTTCCACACGGTTCTTTCAGGCCAAATCACCCCACCCCAACCCACCCCTGGAGATACAGTCAGGAAGAGACTGATGGGGCAGATCCTGGAGGAGGCCGCACTGCCGGAATCTTCCCCTGGCAGAGAAACAGGGCTAAGCAGGCGCCAGGGAGCAAGCCTGGGCTTGCCACATCACCACCTTCTGTGCTGAAGGTCAGATGACACACAGTCACCAGTTTTCCGAGAAGCACAGGGAGGGGTGCATGTCAGGCCCAGCCCTGCCTGCTGATACCACCTCACAGACGCAGGCGAAATGTCTTCACCAGCTTCTTCTCGGTTGCCAAGGGGGCCTGCTTTCAAGGGGTTGTTTTGTATGGGGTGTGGGTGTTAACCAGGGAGAGGTTCCTGGCAGGAGTTCCTGTCAGATGCCATTTTCCATTCTGGTTACTTGAACCGGGGTCCTTTTTCATCCTCTACCTCAGGGGCCAGAGCTTCCTCATCACCCTGCACCCCCTAGCAAGGGAGGTGCCCGCTACACGGTCCAAAGTCCTCTCCAGACATTCGGGCCCTGCCCGCCTTGCTGGAGGGCTCAGGAGTTCACCAGGTTTGCTTCCAGAAGAGCCAGGCTGGGGATGCCTGCTTGCAAATGCAGCAAATAAAAAATAAATAAATAAATAAAAATCACCTTTCTCCCCACCCCCACCCAAATGTAAAATGAGCCGACAGCCTGTCCCTCGGCTATGTCAGATACTGCTTTCCGCACAATATCTTGGTTTTACATTATTTTGCAACGGCCTCTTCCCACTTAATCCATCCTGAACACACTTCTTGAATGTTCTACCCCGGCAGGGATGCGCATTCTCAGGAAACACGTCCCCTCGCCAGGCCCCTGGGAAGAGTCAAAACCACGATGCCTGCCCCTGAAGCGCCCGCGCGGCTCCACGGGGCTCCATGTTGTCACCCACTAGCTCCTGGACGCTATCAGCCCGCAGCAGGGTTTCCTGGCCAGAGAAACCGCCTGTTGGGGTGCGGAGCGTTCCACGTCCCCTCGCAGCACCCCGCTGGCCTTCTGGGCCGCCCCGCCCCCAACCCGCGCCCGGGCGCTGACGTCATCGGCCGAGCCCGACTCGGAACCACCGCCCGAAAGTCACGCGCGCCTCCCGGCGGGACGTGCCCTCTGCAGAGATCCCTCCGCCGCCGCCTGGAATTTTCCAGATGTCCTGCCGGCCCTTCTTTCGGGCCTCGGCTGGGCGTGGTGACCTCAAGCGACCCCGGCGGCTCCCGTCGCCCACTCAAGAGGGCGCCCCCGCCCGCCACGCCCCCAGCCCCTCTCGGGGAAGCGCGTCCCCCGCCCGACCTCAACAACATCCGGGAAGAAAAGGGGTCTTCTTCCTCATGGCATTCGCCTCCCAAATGTCACCTTGAGCGCTGTCCGCGATCCCAAAAAGCACTGTTAAAGAGAAGCGGGGATACACTGGGGTCCTTGCGTGGGGGCCGCCTGGGAGACCATGCACCGCCCGGGAGTCTCTCCGGGCAGGGTCGGGGAGGGACTTTTTTGCCGGTGGCCGTGGAGGAATCGTCCCGTTGAGCAATGACCCCGGTGACGCGGCTGAGGGCTATAAAAGCAAAAGTTGTGGCAAGTCCAGCC
